# You are what you eat: diet, microbiota and mental health

**DOI:** 10.1192/j.eurpsy.2024.1430

**Published:** 2024-08-27

**Authors:** A. M. Matas Ochoa, S. Rubio Corgo, I. Durán Cristóbal

**Affiliations:** Psychiatry, Infanta Leonor University Hospital, Madrid, Spain

## Abstract

**Introduction:**

In recent years, there is a growing interest in microbiota and how certain dietary patterns affect our brain.

We know that diet has an important impact in physical and mental health. The mecanism that underlies is already unknown, but there is emerging evidence that diet modulates brain gut microbiota and has implications in mental problems.

**Objectives:**

The aim of this poster is highlight the importance of diet in mental health and the link with microbiota.

**Methods:**

Review of recent literature about diet, microbiota and psychiatry. The studies were collected of the electronic databases PubMed.

**Results:**

New researches highlight the importance of adequate nutrition for mental health. Several studies link healthy diet with a minor risk of mental illnesses or with the improvement of depressive symptoms. Likewise, poor dietary habits could aggravate cognitive decline and increased risk of developing anxiety, depression or other mental illnesses.

It has been shown that a diet rich in fiber, polyphenols and micronutrients improve gut microbial composition and can reduce metabolic endotoxemia and neuroinflammation, and this has been associated with improvements in brain health. Also, prebiotic and probiotics have positive effects.

Therefore, dietary interventions could be a complementary therapeutic approach for patients with mental problems. This is what nutritional psychiatry focuses on.

**Conclusions:**

Microbiota as a potential therapeutic target for mental illness is a hot topic in psychiatry, but also, its interaction with dietary change or the use of probiotics and prebiotics. This action is easy to implement in our clinical practice and could be part of a biopsychosocial treatment to improve or prevent some psychiatric disorders.

Nutritional psychiatry is a new field that needs to be developed and the knowledge in microbiota, diet and mental health could help. Hopefully, the research about this topic continues expanding.

**Disclosure of Interest:**

None Declared

